# The inevitability of C_4_ photosynthesis

**DOI:** 10.7554/eLife.03702

**Published:** 2014-07-22

**Authors:** Erika J Edwards

**Affiliations:** 1 **Erika J Edwards** is at Brown University, Providence, United Stateserika_edwards@brown.edu

**Keywords:** Flaveria, C4 photosynthesis, evolution, plant evolution, other

## Abstract

Elements of C_4_ photosynthesis—a complex adaptation that increases photosynthetic efficiency—may have evolved first to correct an intercellular nitrogen imbalance, and only later evolved a central role in carbon fixation.

**Related research article** Mallmann J, Heckmann D, Bräutigam A, Lercher MJ, Weber APM, Westhoff P, Gowik U. 2014. The role of photorespiration during the evolution of C_4_ photosynthesis in the genus *Flaveria*. *eLife* **3**:e02478. doi: 10.7554/eLife.02478

**Image** C_4_ plants (Fb and Ft) have higher levels of certain enzymes than plants that use other forms of photosynthesis

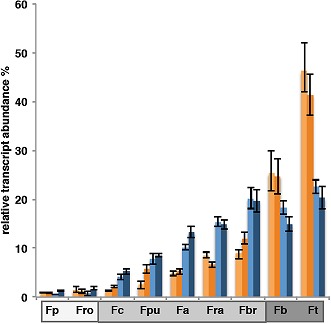

Understanding the evolution of complex innovations remains one of the most challenging problems in biology (Lynch, 2007; Wagner, 2014). Insights often stem from experimental lab studies that manipulate systems under 'directed evolution' (Weinreich et al., 2006; Blount et al., 2012; Finnigan et al., 2012). However, complex traits that have evolved many times over in independent lineages present a different—yet equally powerful—opportunity to infer the evolutionary trajectories of novel traits.

In flowering plants, C_4_ photosynthesis is a well-studied, complex adaptation that has independently evolved over 60 times (Sage et al., 2011). Many key, shared stages along the C_4_ evolutionary trajectory have been identified by studying multiple C_4_-evolving plant groups (e.g., Kennedy et al., 1980; Ku et al., 1983; Vogan et al., 2007; Williams et al., 2013). Now, in *eLife*, Udo Gowik and colleagues at Heinrich-Heine-Universität—including Julia Mallmann and David Heckmann as joint first authors—present a compelling new hypothesis for how the final evolutionary steps were realized (Mallmann et al., 2014).

Although atmospheric carbon dioxide (CO_2_) levels are currently rising, the last 30 million years witnessed great declines in CO_2_, which has limited the efficiency of photosynthesis. Rubisco, the critical photosynthetic enzyme that catalyses the fixation of CO_2_ into carbohydrate, also reacts with oxygen when CO_2_ levels are low and temperatures are high. When this occurs, plants activate a process known as photorespiration, an energetically expensive set of reactions that—importantly for this story—release one molecule of CO_2_.

C_4_ photosynthesis is a clever solution to the problem of low atmospheric CO_2_. It is an internal plant carbon-concentrating mechanism that largely eliminates photorespiration: a 'fuel-injection' system for the photosynthetic engine. C_4_ plants differ from plants with the more typical 'C_3_' photosynthesis because they restrict Rubisco activity to an inner compartment, typically the bundle sheath, with atmospheric CO_2_ being fixed into a 4-carbon acid in the outer mesophyll. This molecule then travels to the bundle sheath, where it is broken down again, bathing Rubisco in CO_2_ and limiting the costly process of photorespiration.

The evolution of the C_4_ pathway requires many changes. These include the recruitment of multiple enzymes into new biochemical functions, massive shifts in the spatial distribution of proteins and organelles, and a set of anatomical modifications to cell size and structure. It is complex, and it is also highly effective: C_4_ plants include many of our most important and productive crops (maize, sorghum, sugarcane, millet) and are responsible for around 25% of global terrestrial photosynthesis (Still et al., 2003).

A key intermediate step in the evolution of C_4_ is the establishment of a rudimentary carbon-concentrating mechanism. Termed 'C_2_ photosynthesis', this mechanism limits certain reactions of the photorespiratory cycle to the bundle sheath cells. A byproduct of these reactions is CO_2_, creating a slightly elevated CO_2_ concentration and increasing Rubisco efficiency in these cells. Though much rarer than C_4_ plants, C_2_ plants have been discovered in a variety of C_4_-evolving lineages, and are thought to represent a common, if not requisite, intermediate step along the C_4_ trajectory (Sage et al., 2012).

One implication of a restricted photorespiratory cycle is the development of a severe nitrogen imbalance between the mesophyll and the bundle sheath cells. This occurs because every molecule of CO_2_ produced in the bundle sheath is accompanied by a molecule of ammonia. While this nitrogen imbalance has previously been recognised (Monson and Rawsthorne, 2000), it has never been closely studied, and certainly never considered as potentially important to the evolutionary assembly of the C_4_ pathway.

To investigate this, Mallmann, Heckmann et al. combined a mechanistic model of C_2_ physiological function with a metabolic model, which allowed them to predict the buildup of certain metabolites based on the rates of Rubisco and photorespiratory activity. They then modelled the various biochemical pathways that could potentially be induced to balance metabolic fluxes between the mesophyll and bundle sheath cells. This creative combination of models allowed them to evaluate the various metabolic pathways for re-balancing nitrogen in terms of which pathways resulted in the highest biomass yield (a proxy for fitness).

Remarkably, when low levels of C_4_ enzyme activity are permitted in the model, key elements of the C_4_ cycle are favoured as the nitrogen-balancing pathway. What's more, this model predicts that with a C_4_ cycle established, increasing the activity of the enzymes results in a linear increase in biomass yield. Allowing for low levels of C_4_ enzyme activity is biologically reasonable, as these enzymes are routinely present in C_3_ leaves. Mallmann, Heckmann et al. support their model predictions with experimental gene expression data from a set of C_3_, C_2_, C_4_, and other C_3_-C_4_ intermediate types in the plant lineage *Flaveria*, which show elevated C_4_ cycle activity even in intermediates that are not using the enzymes to capture carbon.

In other words, once a C_2_ cycle is established, the evolution of a fully realized C_4_ process is fairly trivial. Once C_4_ enzymes are recruited to shuttle nitrogen back to the mesophyll, it is all but inevitable. This can explain in part why C_4_ has evolved such a startling number of times, and why many of these origins are highly clustered across the tree of life. Many C_4_ evolutionary clusters likely share an ancestor that had already acquired an elevated likelihood of evolving the pathway (Figure 1).

**Figure 1.**
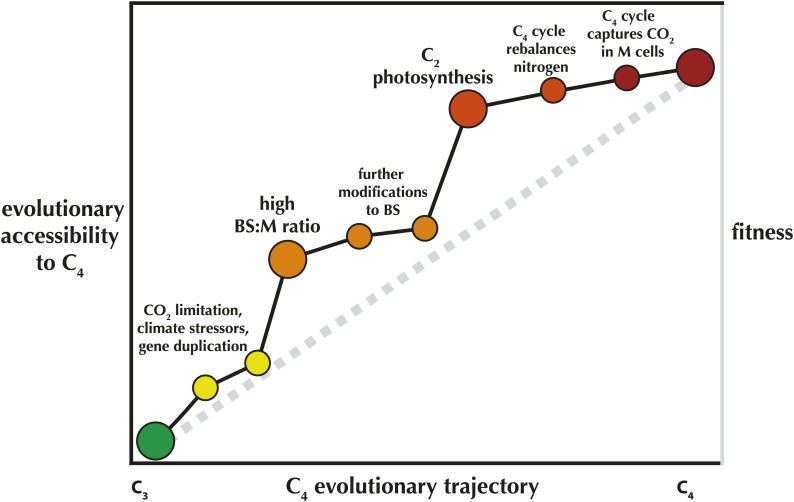
An 'Evolvability Landscape' for C_4_ photosynthesis. Many intermediate stages along the evolutionary trajectory from C_3_ to C_4_ are well known (Sage et al., 2012). These can be displayed as part of an adaptive fitness landscape, which links biological properties (horizontal axis) with the fitness they produce (right vertical axis; a greater height indicates a greater fitness). The adaptive fitness landscape of the C_4_ trajectory was recently modelled as 'Mt. Fuji-like': a steep linear incline with each step along the trajectory bringing small, incremental increases in fitness (Heckmann et al., 2013), represented here by the grey dashed line. The gains in relative likelihood of evolving C_4_, or the 'evolutionary accessibility' of the pathway, may not be so linear (left vertical axis; black line). In spite of some limited flexibility in the order of trait acquisition (Williams et al., 2013), two intermediate stages are relatively fixed in position along the trajectory and also provide steep increases in C_4_ evolvability. One early step, an elevated ratio of bundle sheath: mesophyll cross-sectional area (BS:M ratio) was recently identified as a key trait that preceded multiple parallel realizations of C_4_ (Christin et al., 2013). Mallman et al. propose a mechanistic interaction between C_2_ and C_4_ photosynthesis, suggesting that evolution of the C_2_ stage of the trajectory greatly increases the probability that full C_4_ photosynthesis will quickly follow.

This may also explain why C_2_ species are so rare relative to C_4_ species—C_2_ is likely to be a step along the trajectory with a relatively short evolutionary lifespan. At the same time, it raises the question of why a handful of C_2_ species are persistent—the C_2_ *Mollugo verticillata* group may be up to 15 million years old (Christin et al., 2011). A testable hypothesis would be that these C_2_ plants have solved their nitrogen problem a different way, thereby limiting their own evolutionary accessibility to C_4_ photosynthesis. If so, this highlights the key role of contingency in adaptation, and our growing power to understand and predict macroevolutionary processes.
